# Supplementary material for: Modulation of flight and feeding behaviours requires presynaptic IP3Rs in dopaminergic neurons
Source: eLife. 2020 Nov 6;9:e62297. doi: 10.7554/eLife.62297 (PMC7647402; doi:10.7554/eLife.62297)
Supplement: Figure 1—source data 1. [file elife-62297-fig1-data1.pptx]

## Slide 1
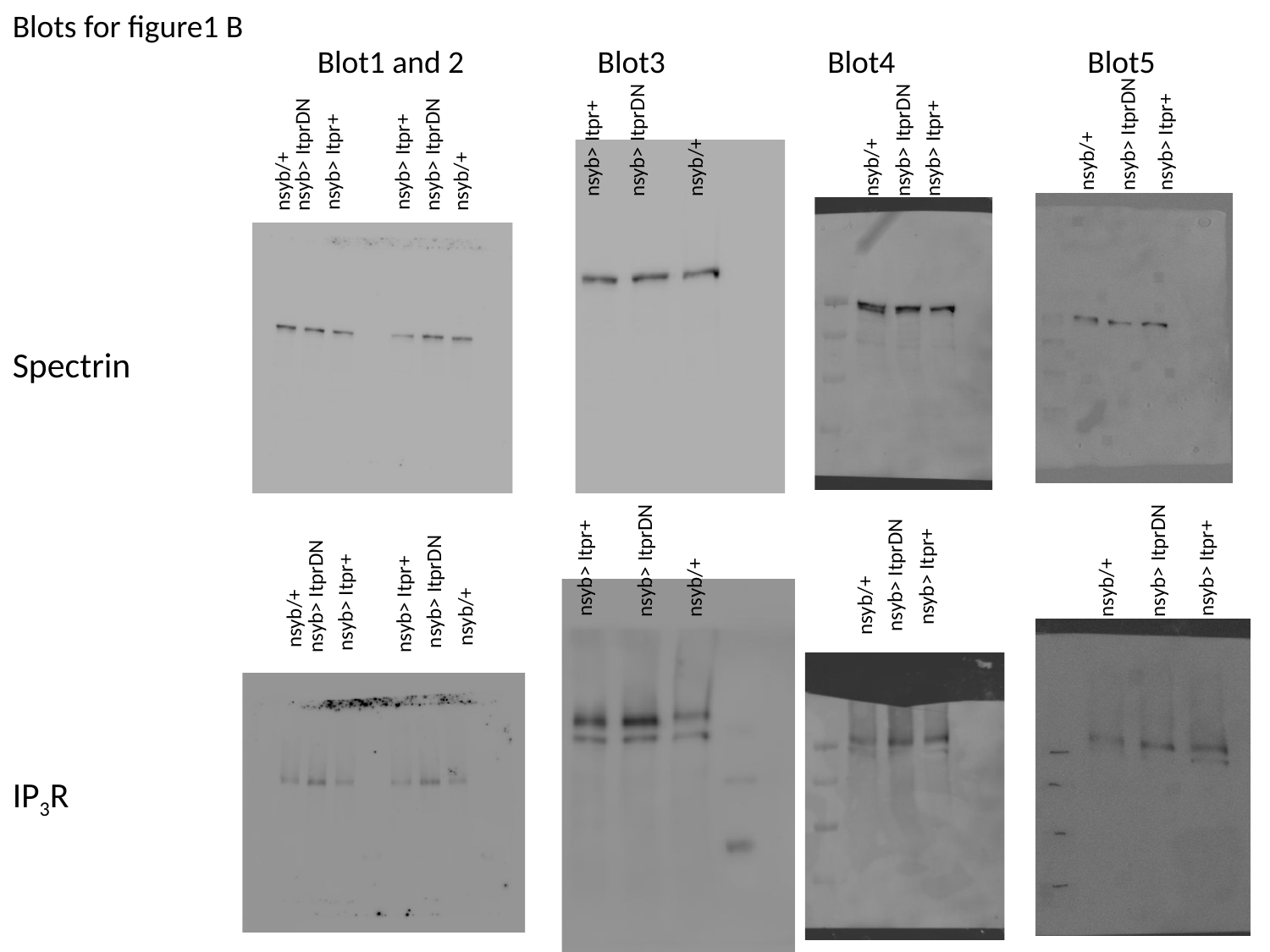

Blots for figure1 B
nsyb> ItprDN
nsyb> Itpr+
nsyb/+
nsyb> ItprDN
nsyb> Itpr+
nsyb/+
nsyb> ItprDN
nsyb> Itpr+
nsyb/+
nsyb> ItprDN
nsyb> Itpr+
nsyb/+
nsyb> ItprDN
nsyb> Itpr+
nsyb/+
nsyb> Itpr+
nsyb> ItprDN
nsyb/+
nsyb> ItprDN
nsyb> ItprDN
nsyb> Itpr+
nsyb> Itpr+
nsyb/+
nsyb/+
nsyb> ItprDN
nsyb> Itpr+
nsyb> ItprDN
nsyb> Itpr+
nsyb/+
nsyb/+
Blot1 and 2
Blot3
Blot4
Blot5
Spectrin
IP3R
