# Supplementary material for: Modulation of flight and feeding behaviours requires presynaptic IP3Rs in dopaminergic neurons
Source: eLife. 2020 Nov 6;9:e62297. doi: 10.7554/eLife.62297 (PMC7647402; doi:10.7554/eLife.62297)
Supplement: Figure 3—source data 3. [file elife-62297-fig3-data3.pptx]

## Slide 1
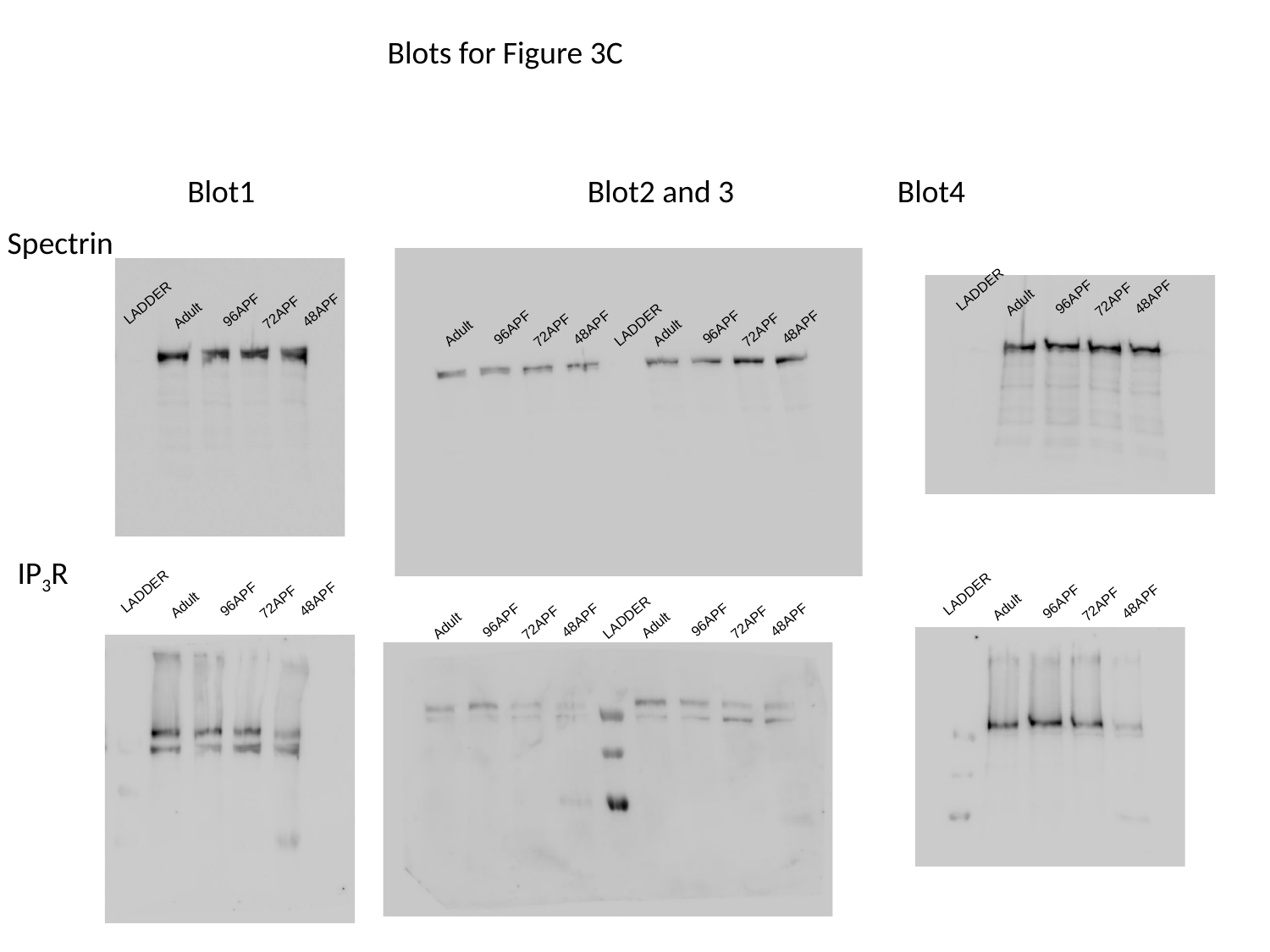

Blots for Figure 3C
Blot1
Blot2 and 3
Blot4
Spectrin
LADDER
96APF
48APF
72APF
Adult
IP3R
LADDER
96APF
48APF
72APF
Adult
LADDER
96APF
48APF
96APF
48APF
72APF
72APF
Adult
Adult
LADDER
96APF
48APF
72APF
Adult
LADDER
96APF
48APF
72APF
Adult
LADDER
96APF
48APF
72APF
Adult
LADDER
96APF
48APF
96APF
48APF
72APF
72APF
Adult
Adult
