# Supplementary figures and images for: Modulation of flight and feeding behaviours requires presynaptic IP3Rs in dopaminergic neurons
Source: eLife. 2020 Nov 6;9:e62297. doi: 10.7554/eLife.62297 (PMC7647402; doi:10.7554/eLife.62297)

## Slide 1
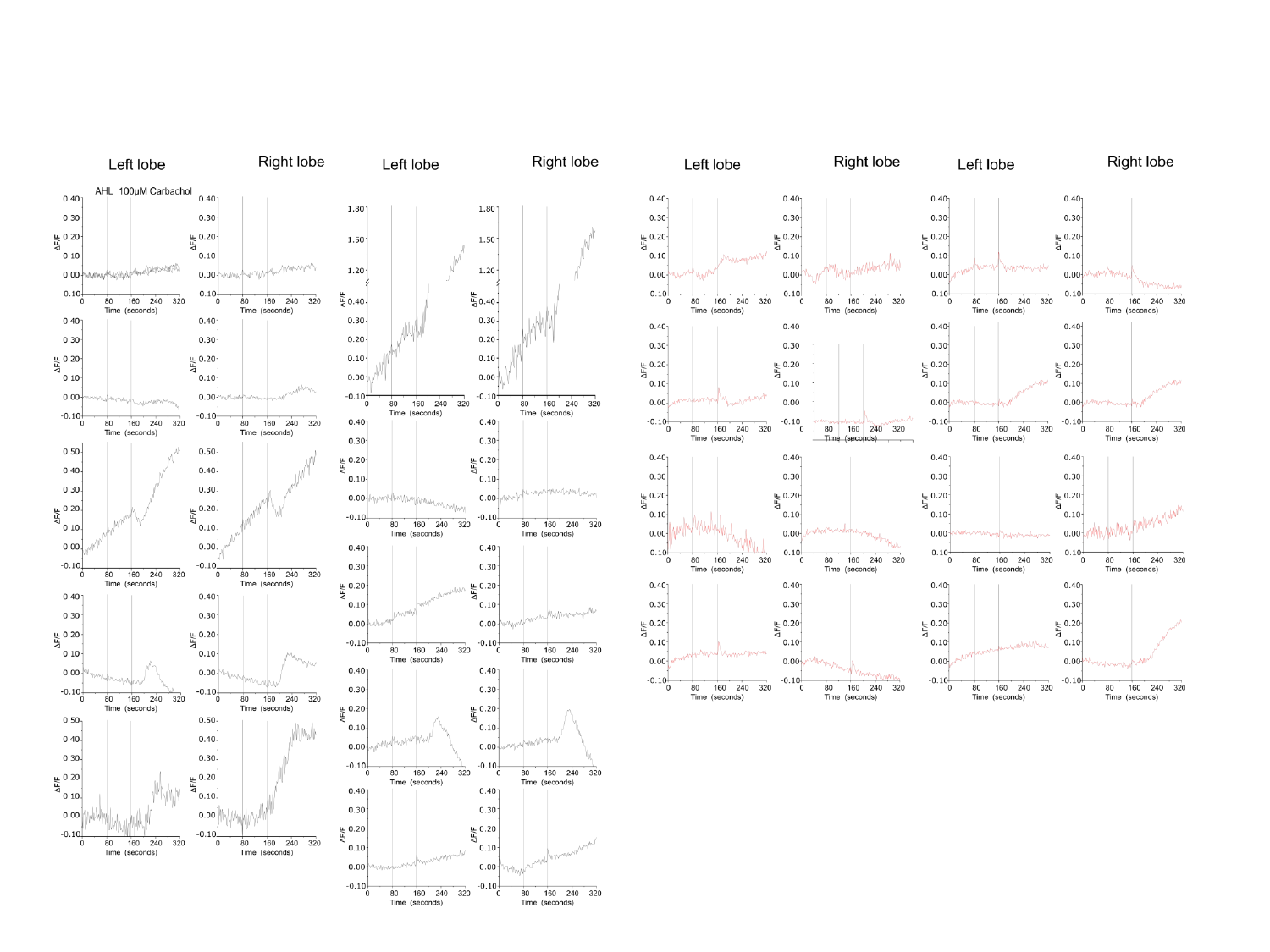

Supplement: Figure 5—source data 1. [file elife-62297-fig5-data1.pptx]

## Slide 1
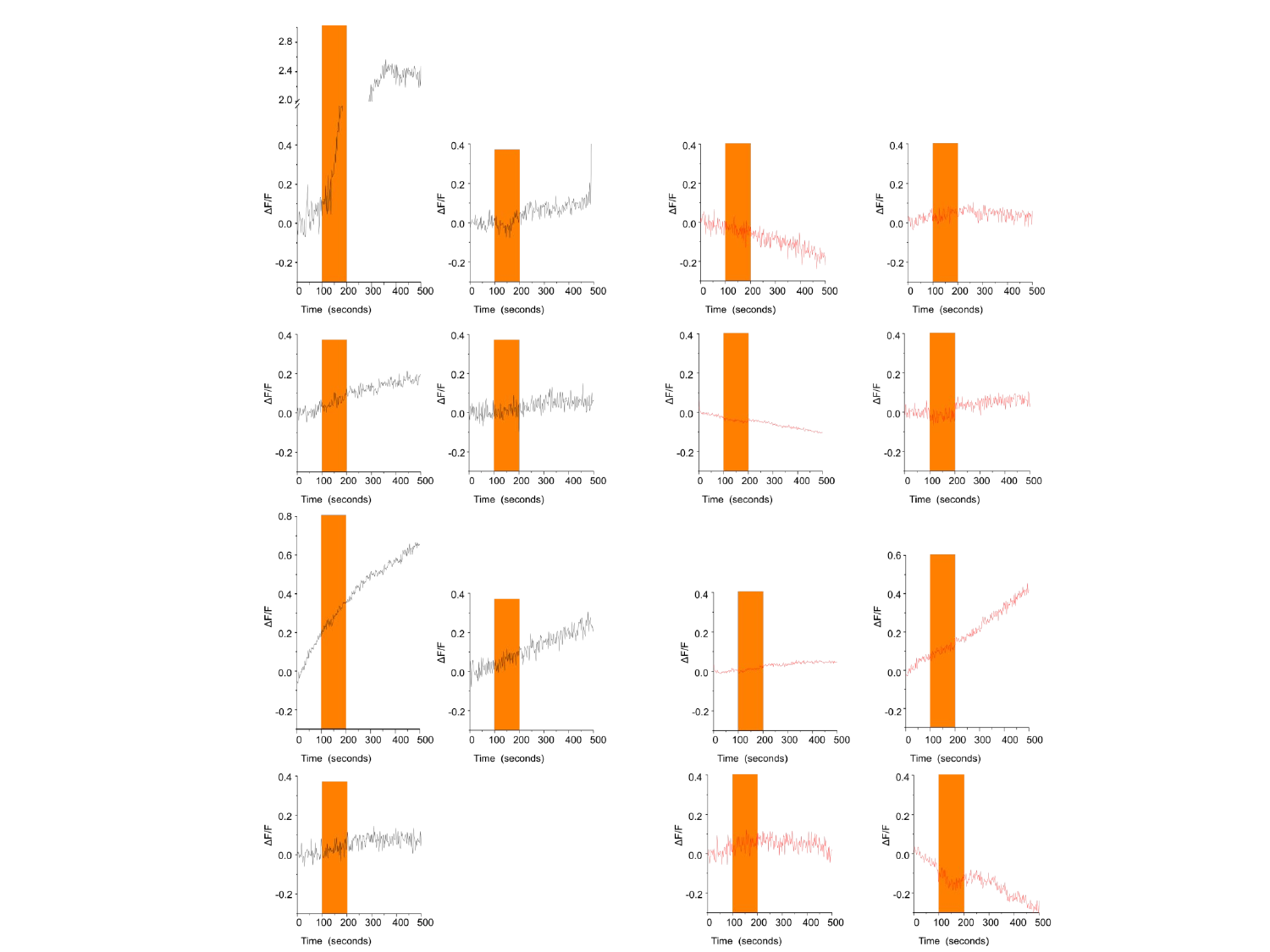

Supplement: Figure 6—source data 1. [file elife-62297-fig6-data1.pptx]

## Slide 1
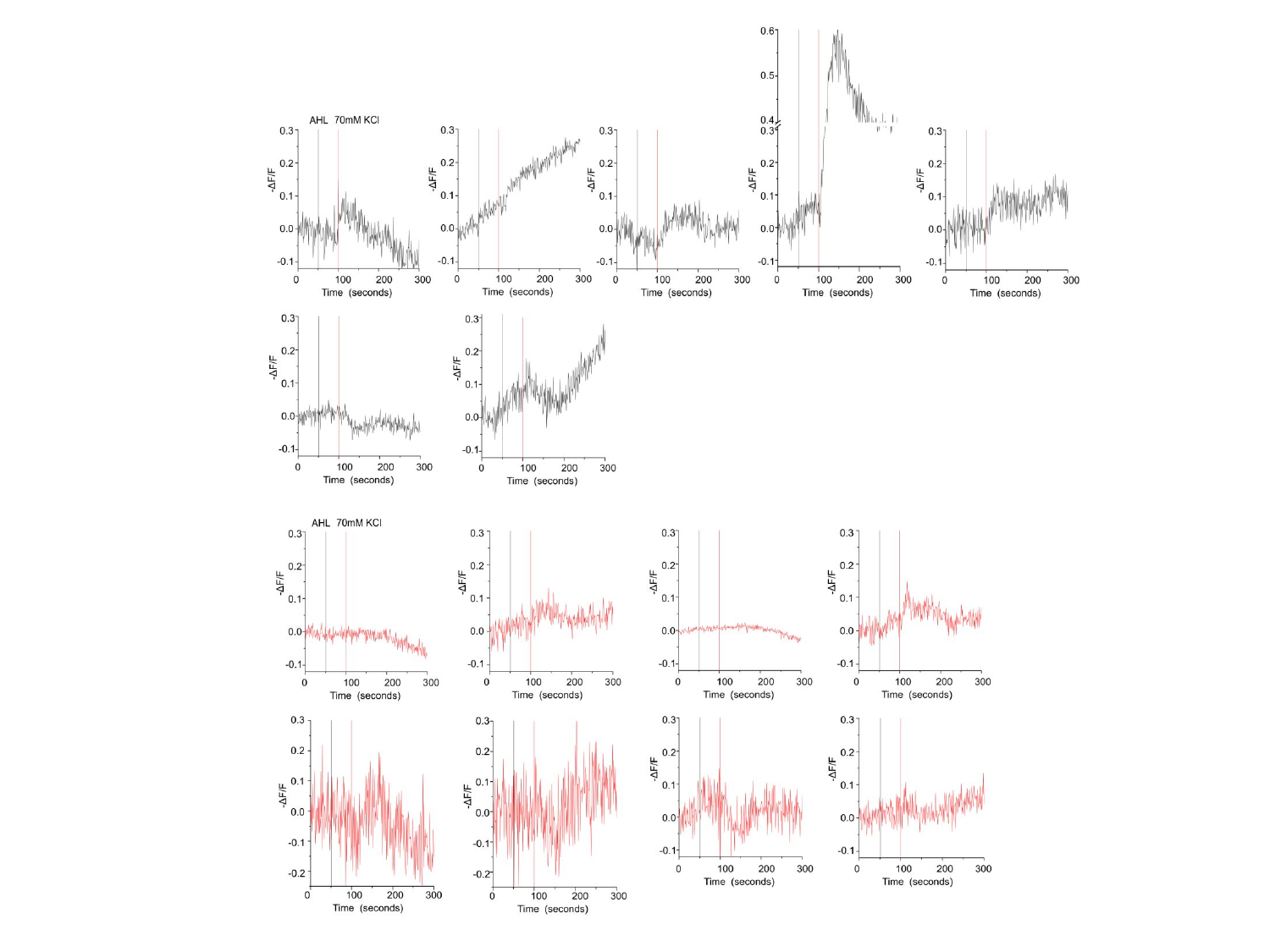

Supplement: Figure 6—source data 2. [file elife-62297-fig6-data2.pptx]

## Slide 1
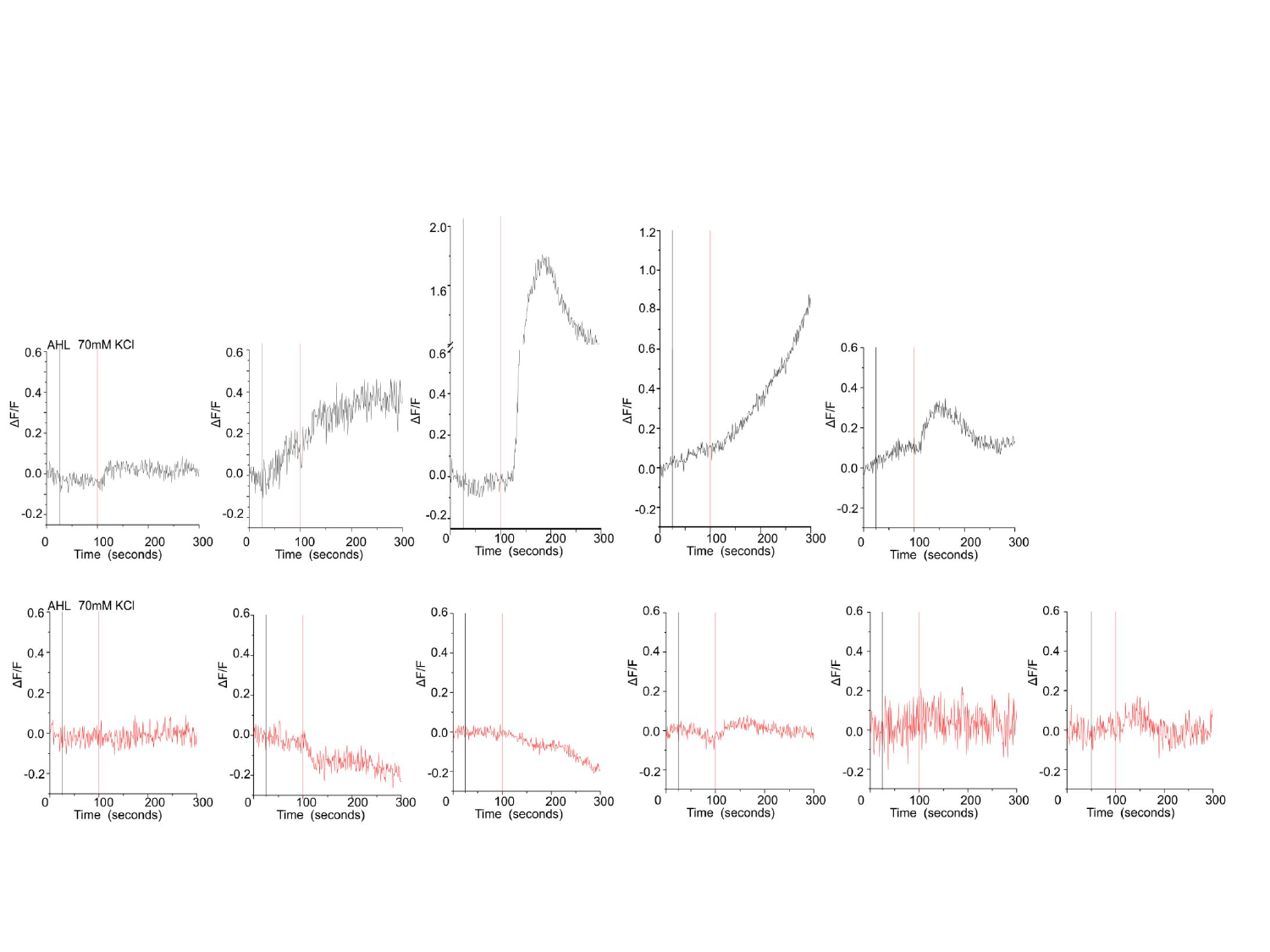

Supplement: Figure 6—source data 3. [file elife-62297-fig6-data3.pptx]
