# Supplementary material for: Modulation of flight and feeding behaviours requires presynaptic IP3Rs in dopaminergic neurons
Source: eLife. 2020 Nov 6;9:e62297. doi: 10.7554/eLife.62297 (PMC7647402; doi:10.7554/eLife.62297)
Supplement: Transparent reporting form [file elife-62297-transrepform.docx]

***eLife’s* transparent reporting form**

We encourage authors to provide detailed information *within their submission* to facilitate the interpretation and replication of experiments. Authors can upload supporting documentation to indicate the use of appropriate reporting guidelines for health-related research (see [EQUATOR Network](http://www.equator-network.org/%20)), life science research (see the [BioSharing Information Resource](https://biosharing.org/)), or the [ARRIVE guidelines](http://www.plosbiology.org/article/info:doi/10.1371/journal.pbio.1000412) for reporting work involving animal research. Where applicable, authors should refer to any relevant reporting standards documents in this form.

If you have any questions, please consult our Journal Policies and/or contact us: [editorial@elifesciences.org](mailto:editorial@elifesciences.org).

**Sample-size estimation**

- You should state whether an appropriate sample size was computed when the study was being designed
- You should state the statistical method of sample size computation and any required assumptions
- If no explicit power analysis was used, you should describe how you decided what sample (replicate) size (number) to use

Please outline where this information can be found within the submission (e.g., sections or figure legends), or explain why this information doesn’t apply to your submission:

No sample size computations were made.

For flight assays, sample size of > 25 was tested as this number was sufficient to yield statistically significant and reproducible results in previous studies (Manjila et al., 2019, Current Biology; Pathak et al., 2015, J Neuroscience)

Similarly, N=30 for the food seeking assay was based on a protocol for this assay obtained from Tsao et al., 2018, elife.

For western blots, N ≥ 3 biological samples while for imaging experiments N ≥ 5 biological samples were used as these are the numbers normally used for such experiments (Pathak et al., 2015, J Neuroscience; Berry et al., 2018, Cell Reports) and then an appropriate statistical test was used to test for significance. Statistical tests are mentioned in the figure legends.

**Replicates**

- You should report how often each experiment was performed
- You should include a definition of biological versus technical replication
- The data obtained should be provided and sufficient information should be provided to indicate the number of independent biological and/or technical replicates
- If you encountered any outliers, you should describe how these were handled
- Criteria for exclusion/inclusion of data should be clearly stated
- High-throughput sequence data should be uploaded before submission, with a private link for reviewers provided (these are available from both GEO and ArrayExpress)

Please outline where this information can be found within the submission (e.g., sections or figure legends), or explain why this information doesn’t apply to your submission:

For flight assays three or more experiments were performed. Each experiment was with 7-10 flies of each genotype aged 3-5 days.

Similarly, for the food seeking assay, 3-5 experiments were performed with 5-7 males of each genotype.

Total number of animals used for each assay is mentioned in the figure legends.

For western blots, 3-5 biological replicates for each genotype were tested. Exact numbers are given in the figure legends.

For imaging experiments, 5 or more brains were imaged for each genotype. Exact number of brains and number of cells imaged are given in figure legends

**Statistical reporting**

- Statistical analysis methods should be described and justified
- Raw data should be presented in figures whenever informative to do so (typically when N per group is less than 10)
- For each experiment, you should identify the statistical tests used, exact values of N, definitions of center, methods of multiple test correction, and dispersion and precision measures (e.g., mean, median, SD, SEM, confidence intervals; and, for the major substantive results, a measure of effect size (e.g., Pearson's r, Cohen's d)
- Report exact p-values wherever possible alongside the summary statistics and 95% confidence intervals. These should be reported for all key questions and not only when the p-value is less than 0.05.

Please outline where this information can be found within the submission (e.g., sections or figure legends), or explain why this information doesn’t apply to your submission:

Statistical tests and N per group are reported in the individual figure legends.

Also source file is provided for exact p values

(For large datasets, or papers with a very large number of statistical tests, you may upload a single table file with tests, Ns, etc., with reference to sections in the manuscript.)

**Group allocation**

- Indicate how samples were allocated into experimental groups (in the case of clinical studies, please specify allocation to treatment method); if randomization was used, please also state if restricted randomization was applied
- Indicate if masking was used during group allocation, data collection and/or data analysis

Please outline where this information can be found within the submission (e.g., sections or figure legends), or explain why this information doesn’t apply to your submission:

It is not applicable to our study as each experimental group had samples picked from a single cross and no bias was assigned while selecting flies for any behavioral assay or other experiments.

**Additional data files (“source data”)**

- We encourage you to upload relevant additional data files, such as numerical data that are represented as a graph in a figure, or as a summary table
- Where provided, these should be in the most useful format, and they can be uploaded as “Source data” files linked to a main figure or table
- Include model definition files including the full list of parameters used
- Include code used for data analysis (e.g., R, MatLab)
- Avoid stating that data files are “available upon request”

Please indicate the figures or tables for which source data files have been provided:

An excel file is attached as source data for raw data used to plots graphs for flight assays as well as food seeking assays.

Imaging files are very large in size and can be provided, if required. But traces for fluorescence change is provided for every cell

Powerpoint file is provided showing all the western blots performed.

Source file for statistics is provided
